# Trends and Gaps in Colorectal Cancer Screening Research in the Arab World: A 16-Year Bibliometric Analysis (2007–2023)

**DOI:** 10.3390/ijerph22020264

**Published:** 2025-02-12

**Authors:** Noura Abbas, Laudy Chehade, Hawraa Tarhini, Zahi Abdul Sater, Ali Shamseddine

**Affiliations:** 1 Naef K. Basile Cancer Institute, American University of Beirut Medical Center, Riad El Solh, Beirut 1107-2020, Lebanon; na331@aub.edu.lb (N.A.); lc30@aub.edu.lb (L.C.); ht52@aub.edu.lb (H.T.); 2 College of Public Health, Phoenicia University, Mazraat El Daoudiyeh 1600, Lebanon; zahi.adulsater@pu.edu.lb

**Keywords:** colorectal cancer, screening, bibliometric, Arab world, colonoscopy, awareness, research gaps, country production, implementation

## Abstract

Colorectal cancer (CRC) is a significant public health concern, ranking third in incidence and second in mortality worldwide. Despite rising CRC incidence rates in the Arab world, understanding of trends and patterns in CRC screening research remains limited. This study addresses this gap through a bibliometric analysis of CRC screening research in the Arab world from 2007 to 2023. We conducted an extensive literature search in Web of Science and Scopus databases, analyzing 124 articles using the Bibliometrix Package in R. Our findings revealed a 16.5% annual growth in research output, with significant increases from 2014 onwards. Saudi Arabia led in scientific production, followed by Lebanon, Jordan, and Egypt, while Qatar had the highest country production when adjusted for population size. Disparities in research output relative to the CRC burden were evident, especially in lower-resource countries. Three regional clusters were identified: Saudi Arabia, with strong collaborations with Canada and Egypt; a second cluster including Lebanon, UAE, Jordan, Qatar, Iraq, and Oman; and a third cluster featuring Morocco, with significant collaboration with France. Thematic analysis showed a focus on CRC screening awareness, barriers, and adherence but a lack of studies on implementation strategies and cost-effectiveness. This analysis highlights significant trends and gaps in CRC screening research in the Arab world, underscoring the need for increased investment in CRC research and screening initiatives to improve outcomes in the region.

## 1. Introduction

Colorectal cancer (CRC) ranks as the third most common cancer globally and the second leading cause of cancer-related deaths, according to the most recent GLOBOCAN estimates [1]. Over the past decade, the incidence of CRC in the Arab world has been on the rise. Despite the available literature, there are no data on the overall cumulative incidence and mortality of CRC in the Arab world. A systematic review by Makhlouf et al. [2] reported the prevalence of CRC in specific Arab countries. The prevalence of CRC in Egypt varied from 0.4 to 14%, while Saudi Arabia reported a prevalence of 0.72%.

Data from GLOBOCAN for the years 2012, 2018, and 2020 were used to analyze the CRC incidence and mortality over these years in the Arab world [3,4,5]. Among Arab countries, the Gaza Strip reported the highest age-standardized incidence rate (ASIR) (18.2 per 100,000), while Comoros had the lowest ASIR (3.65 per 100,000). Overall, there has been a multi-fold increase in ASIR in the Arab countries compared to the ASIR previously reported on GLOBOCAN. For instance, the incidence of CRC in Gulf Cooperative Council (GCC) countries showed an upward trend, except for Kuwait, which had a stable incidence. Considering both genders among Arab countries, Libya reported the highest age-standardized mortality rate (ASMR) (11.74 per 100,000), while Comoros and Egypt had the lowest estimated ASMR (2.58 and 3.36 per 100,000, respectively) [1].

Despite the availability of epidemiological data, CRC survival rates in Arab countries remain lower than in Western countries, largely due to the limited implementation of effective screening programs [6]. The implementation of CRC screening strategies has helped reduce the incidence and mortality rates of the disease by approximately 30% in the United States among adults aged 50 and older in the past fifteen years. The effectiveness of these strategies is attributed not only to their ability to facilitate early detection of the disease but also to their capacity to enable the removal of precursor lesions [7,8]. However, the landscape of CRC screening practices in the Arab world remains poorly understood. Although there has been some research on CRC incidence and prevalence, there is a critical gap in understanding the trends and patterns of research focused on CRC screening in this region [8].

To address this gap, we conducted a bibliometric analysis to examine the trends in knowledge production related to CRC screening in the Arab world. This analysis included studies published on CRC screening from 2007 to 2023 (inclusive) in the following Arab countries: Algeria, Bahrain, Comoros, Djibouti, Egypt, Iraq, Jordan, Kuwait, Lebanon, Libya, Mauritania, Morocco, Oman, Palestine (Gaza Strip and West Bank), Qatar, Saudi Arabia, Somalia, Sudan, Syria, Tunisia, United Arab Emirates (UAE), and Yemen. This analysis aimed to understand the patterns and trends in research production on CRC screening in these Arab countries to identify gaps and needs in this area of research.

## 2. Materials and Methods

### 2.1. Source of Data

We conducted an extensive literature search to identify relevant publications in English from 1 January 2007 to 31 December 2023 that address the topic of CRC screening in the Arab world, allowing us to capture research trends over the past 16 years. For this purpose, we conducted a search across two bibliographic databases: Web of Science and Scopus. Data on demographic indicators for Arab countries and income levels were retrieved from the World Bank’s DataBank [9], while data on the incidence and mortality of CRC were obtained from GLOBOCAN 2022 [1].

### 2.2. Search Strategy

We used a combination of the following keywords, linked by the Boolean operators “OR” and “AND”: “Algeria”, “Bahrain”, “Egypt”, “Iraq”, “Jordan”, “Kuwait”, “Lebanon”, “Yemen”, “Aden”, “Sanaa”, “UAE”, “Abu Dhabi”, “Dubai”, “Libya”, “Morocco”, “Oman”, “Comoros”, “Palestine”, “Gaza”, “West Bank”, “Qatar”, “Saudi Arabia”, “KAS”, “Syria”, “Tunisia”, “Sudan”, “Djibouti”, “Somalia”, “Mauritania”, “Colonic Cancer”, “Rectal Cancer”, “Colorectal Cancer”, “Screening”, “Fecal occult blood test”, “FOBT”, “Colonoscopy”, “Sigmoidoscopy”, “Fecal immunochemical test”, and “FIT”. The search strategy incorporated the use of internal and external truncation, as well as proximity operators, to broaden the search and ensure the inclusion of relevant studies. The detailed search strategy in each database is included in Supplementary Tables S1 and S2.

### 2.3. Inclusion Criteria

We included original and early access articles that focused on the topic of CRC screening in one or more Arab countries, published in English, from 1 January 2007 to 31 December 2023. Review articles, abstracts, and meeting reports were excluded. From the resulting dataset, we excluded articles focusing solely on palliative care, disease burden, or cancer treatment.

### 2.4. Data Management and Selection Process

The metadata of the articles generated by the search strategy in each database was exported into an Excel file. After combining the articles and removing duplicates, two reviewers independently screened the titles and abstracts of the retrieved publications. Any discrepancies were resolved through discussion with a third reviewer. A full-text review of all included references was also independently conducted by the two reviewers to decide on the inclusion or exclusion of each article. After selecting eligible articles from the Web of Science and Scopus databases, we conducted a rough search of the literature to further examine the validity of our search and ensure that the relevant articles were included in the bibliometric analysis.

### 2.5. Data Extraction and Analysis

The final dataset of articles was then analyzed using the Bibliometrix Package (version 4.3.2, K-Synth Srl, Naples, Italy) and the R statistical software package (version 4.2.3, The R Foundation for Statistical Computing, Vienna, Austria) for science mapping analysis. The analysis provided insights on research output on CRC screening in the Arab world, including (a) annual scientific production and article citation; (b) most relevant affiliations; (c) journals in which researchers published; (d) country-specific production; (e) country collaboration patterns; and (f) author’s keywords co-occurrence. The analyzed data were then exported from R and transformed into a graphical format using Flourish (accessible through https://www.bibguru.com/b/how-to-cite-flourish/ (accessed on: 5 December 2024)).

### 2.6. Ethical Approval

This study did not require approval from an Institutional Review Board since the information was publicly available and did not involve interactions with human participants.

## 3. Results

### 3.1. Document Analysis

A total of 934 articles were retrieved from Web of Science and Scopus between 2007 and 2023. Out of these, 124 documents were included in the final analysis (Table 1). Seven hundred and seventy-four articles were unrelated to the topic of CRC screening or the Arab World, and 36 articles were duplicates.

**Figure 1:**
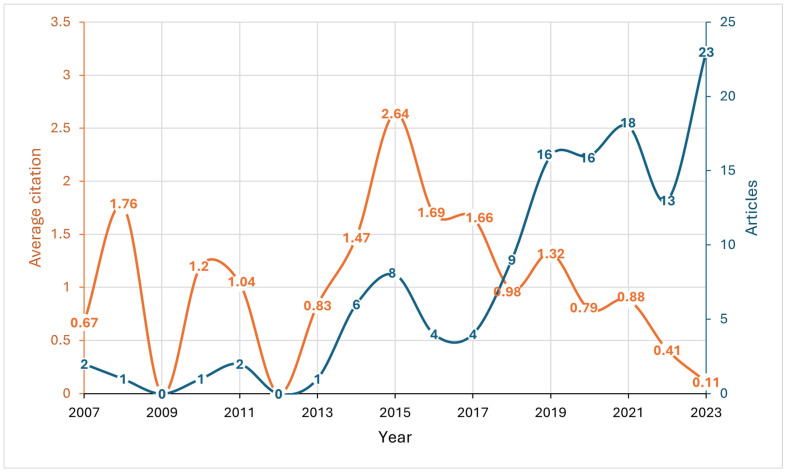
Annual scientific production and article citation on colorectal cancer screening in the Arab world from 2007 to 2023.

The average annual growth rate, defined as the percentage change of research output within a year, was 16.5%. The number of annual publications has seen a significant increase since 2013 (*p*-value < 0.0001 for the overall trend), rising from less than two in previous years to eight in 2015, then decreasing by 50% in 2016 and 2017 before showing an upward trend in 2019 onwards (Figure 1). The average annual citation rate was consistently low throughout the years, ranging from 0.67 in 2007 to a peak of 2.64 in 2015, with a median value of 1.04. The average number of citations per document was 7 (Table 1).

The expression “Author Keywords” refers to the terms or phrases selected by the authors of a research paper to express its essence. They play a role in article indexing and visibility. In contrast, “Keywords Plus” refers to terms generated by a special algorithm that identifies words or phrases frequently appearing in the titles of articles cited by the paper.

### 3.2. Analysis of the Sources

**Figure 2:**
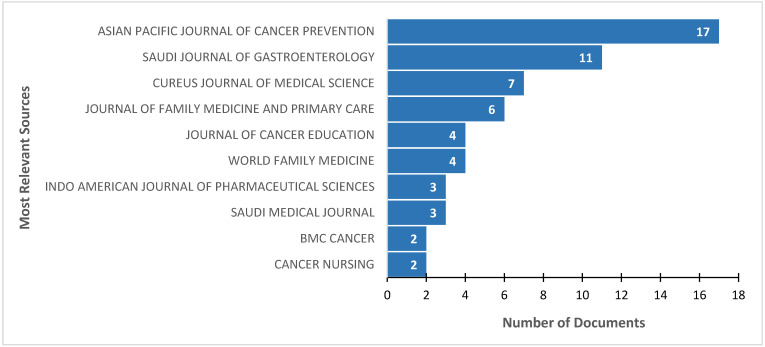
Top 10 most relevant sources based on the number of documents published on colorectal cancer screening in the Arab world from 2007 to 2023.

The articles included in this analysis were published in 70 sources. The *Asian Pacific Journal of Cancer Prevention* (impact factor (IF) 2.5, 17 publications) was the most frequent source of publication, with 24.3% of the articles published in this journal. The second most common journal was the *Saudi Journal of Gastroenterology* (IF 2.7, 11 publications, 15.71%), followed by the *Cureus Journal of Medical Science* (IF 1.2, 7 publications, 10%), the *Journal of Family Medicine and Primary Care* (IF 1.4, 6 publications, 8.57%), and the *Journal of Cancer Education* (IF 1.6, 4 publications, 5.71%) (Figure 2). All these sources are Quartile 3 journals, except for the *Journal of Family Medicine and Primary Care*, which is Quartile 4. The first 10 most frequent journals included almost half of all articles (47.58%), and four of them were regional/local, while six were global.

### 3.3. Analysis of Countries

**Figure 3:**
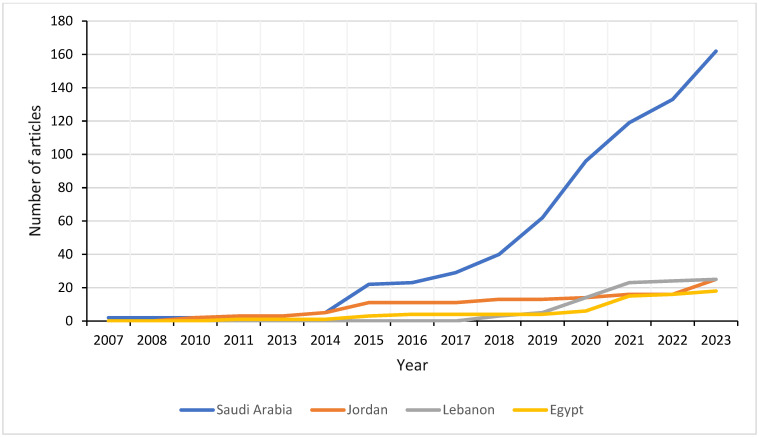
Country production over time of the four most-producing countries of colorectal cancer screening research in the Arab world from 2007 to 2023.

**Figure 4:**
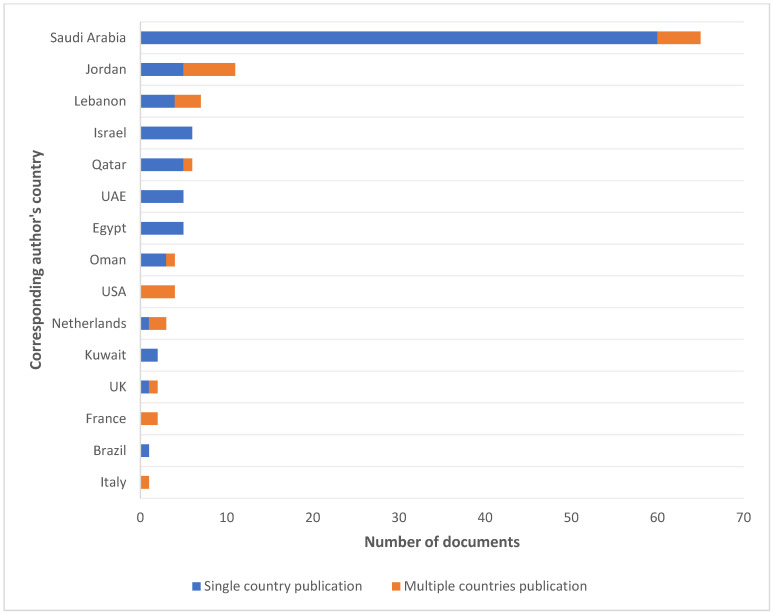
Affiliation countries of the corresponding authors for the 124 documents included in the final analysis, with the relative frequency of single and multiple country publications.

Saudi Arabia is the most prominent country of affiliation (162 affiliations, 46.82%), followed by Lebanon (25 affiliations, 7.22%), Jordan (25 affiliations, 7.22%), and Egypt (18 affiliations, 5.20%) (Figure 3). Most of the corresponding authors were affiliated with Saudi Arabia, and 92% of the articles from this country are single-country publications. This was followed by Jordan (45.4% single-country publications), Lebanon (57.1% single-country publications), Qatar (83% single-country publications), and UAE and Egypt (100% single-country publications) (Figure 4).

**Figure 5:**
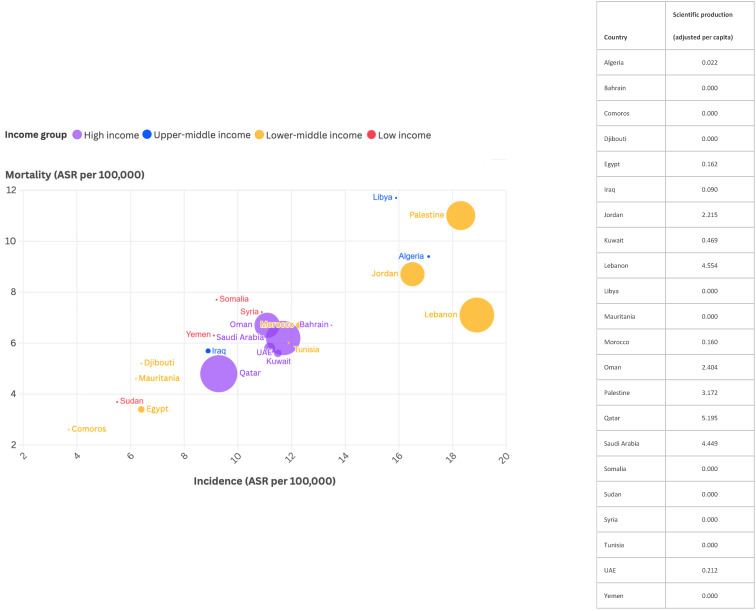
Scatter plot depicting country-specific per capita production of colorectal cancer screening research in Arab countries, along with corresponding incidence and mortality rates. Circle sizes indicate country production adjusted per capita, circle colors reflect the income status of each country, and the x-axis and y-axis represent the incidence and mortality rates (ASR per 100,000) of colorectal cancer, respectively.

When adjusting the country-specific production per capita for each country, Qatar emerged as first (with a country production of 5.2 per million), followed by Lebanon (4.6 per million) and Saudi Arabia (4.4 per million). Moreover, countries with high CRC incidence and mortality did not necessarily have the highest production of articles on CRC screening. For example, Jordan had a high incidence but relatively low production, while Algeria and Libya had no production at all despite elevated incidence and mortality rates [1,9]. Notably, there was no production in low-income countries such as Somalia, Syria, and Sudan. In contrast, Saudi Arabia and Qatar, classified as high-income countries, had high levels of scientific production that corresponded with the incidence of the disease in these countries (Figure 5). Regarding each country’s production over time, Saudi Arabia witnessed a sharp rise starting in 2014–2015 (with an increase from 5 to 22 articles to reach 162 articles in 2023), compared to the other countries, which had a more gradual increase over the years (Figure 3).

### 3.4. Analysis of Authors, Affiliations, and Collaborations

The total number of authors from all 124 documents was 786. On average, there were six authors per document, and only three articles were single-authored—22.58% of co-authorships were international (Table 1).

**Figure 6:**
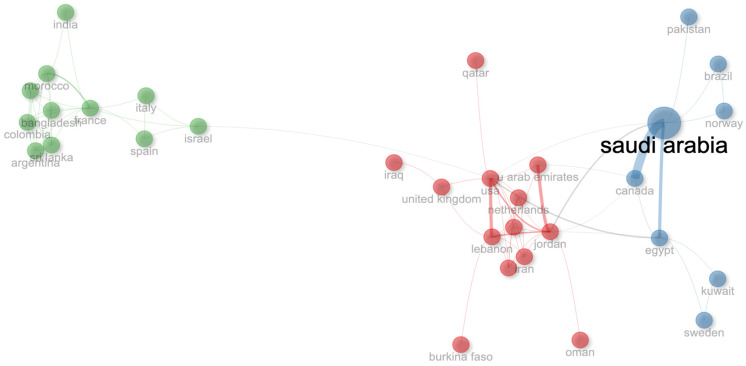
Country collaboration on colorectal cancer screening research in the Arab world from 2007 to 2023. The size of the font and the diameter of the circles represent the number of publications from each country in the dataset. The colors of the circles represent the different clusters or groups of collaborating countries. The thickness of the connecting lines indicates the strength of collaboration between the countries.

Research collaborations based on the authors’ country of affiliation are highlighted in Figure 6. Three distinct clusters are visible, representing regional research alliances. Saudi Arabia stood out as a leading contributor in its cluster, with strong collaboration efforts, mainly with Canada and Egypt. The second cluster regrouped Lebanon, UAE, Jordan, Qatar, Iraq, and Oman, among other non-Arab countries, all as equal contributors. The main collaborations in this cluster were observed between Jordan and UAE, Lebanon and USA, and Lebanon and Jordan. The third cluster included Morocco, among other non-Arab countries, and the strongest collaboration was between Morocco and France.

King Saud University was the most frequent affiliation (44 authors, 5.6%), followed by the American University of Beirut (20, 2.54%), King Abdulaziz University (20, 2.54%), Alexandria University (17, 2.16%), and King Saud Bin Abdulaziz University for Health Science (17, 2.16%) (Supplementary Figure S1). The collaborations between the different institutions contributing to research on CRC screening in the Arab world are illustrated in Supplementary Figure S2. There were eight different clusters, and the most prominent collaborations were between Saudi institutions (mainly King Saud University, King Abdulaziz University, and King Saud Bin Abdulaziz University for Health Science) and Egyptian institutions (Alexandria University and Egyptian Knowledge Bank).

Zubaidi et al. (2015) [10] was the most cited article, with a total of 49 citations, followed by Alsanea et al. (2015) [11] (43 citations), Elsafi et al. (2015) [12] (39 citations), Tfaily et al. (2019) [13] (34 citations), and Azaiza et al. (2008) [14] (30 citations) (Supplementary Figure S3). However, the ranking changes when adjusting for the total number of citations per year to account for the age of the document. In that case, Tfaily et al. (2019) [13] lead with 5.7 citations per year, followed by Zubaidi et al. (2015) [10] (4.9 citations per year), Al-Kuwari et al. (2021) [15] (4.75 citations per year), Alsanea et al. (2015) [11] (4.3 citations per year), and Elsafi et al. (2015) [12] (3.9 citations per year).

The majority of the top 10 most highly cited articles were published in Quartile 3 journals with an IF between 1.6 and 2.9. Two articles from the top 10 were published in Quartile 2 journals with an IF of 3.8 and 4.2, respectively. Five of the top 10 articles were published in local or regional journals, including three from Saudi Arabia and one from Qatar, and the other five were published in global journals. Of note, global journals tended to have a higher IF (average 3.1) compared to local/regional journals (average 2.4).

### 3.5. Analysis of Keywords

**Figure 7:**
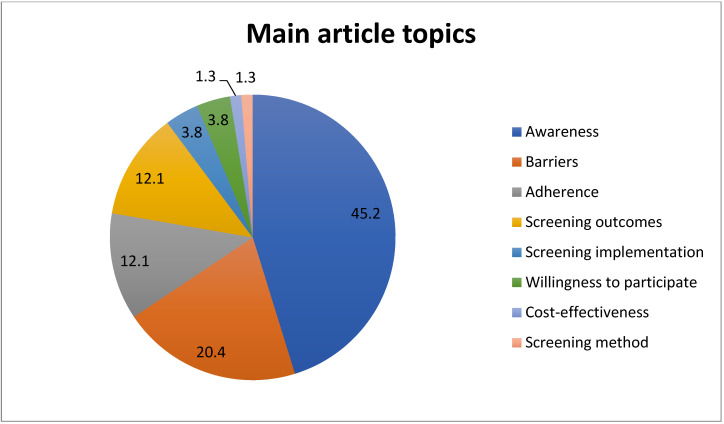
Distribution of the main topics in colorectal cancer screening articles.

Overall, 45% of the articles tackled the topic of CRC screening awareness, which includes knowledge of CRC, its risk factors, and screening. 21% were focused on barriers to screening, 12% on factors influencing adherence to screening guidelines, and 12% on screening outcomes (cancer detection rates of screening tests) (Figure 7).

**Figure 8:**
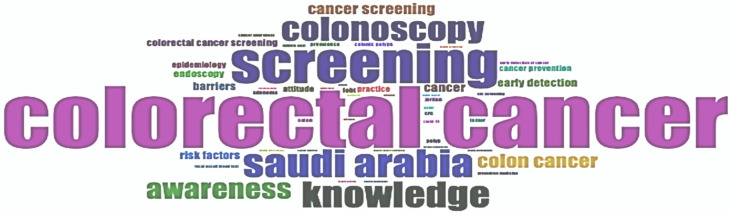
Word cloud of predominant Author Keywords in the colorectal cancer screening literature from the Arab world between 2007 and 2023. The size of the keywords reflects their frequency of occurrence.

An analysis of the authors’ keywords was conducted to understand the knowledge base on CRC screening in the Arab world. A total of 242 distinct keywords were identified (Table 1). The most frequent keywords were “colorectal cancer” (62 times), “screening” (40 times), “knowledge” (26 times), “colonoscopy” (24 times), “awareness” (20 times), “colon cancer” (14 times), “cancer screening” (11 times), “cancer” (9 times), and “barriers” (8 times), and these frequencies are reflected in the word cloud (Figure 8). The co-word network represents the frequency and connection between the different keywords. The most frequent one was “colorectal cancer”, and it seemed to be closely related to the term “knowledge” (Supplementary Figure S4). Other related words were “colonoscopy” and “Saudi Arabia”, which was expected given that this country had the most scientific production on the topic. Supplementary Table S3 summarizes the different themes of the most occurring keywords among the included articles. The most frequent themes were “CRC and CRC screening” (30.6%), followed by “Awareness and attitude” (16.2%). Other common themes included “Country or population” (13.0%), “Methods of screening” (8.9%), “Cancer epidemiology” (6.9%), and “Healthcare and health behavior” (6.9%).

## 4. Discussion

To the best of our knowledge, this is the first bibliometric analysis of CRC screening in the Arab world. The increase in the annual growth rate indicates a growing interest in this topic. However, the impact of these studies within the scientific community may be limited, as evidenced by the low average number of annual citations per document with a median of 1.04 (Figure 1). Among the top 10 most cited articles, four were published in 2015, which explains the peak of average citations per year observed in 2015 (with a value of 2.64) (Figure 1, Supplementary Figure S3). Additionally, most of the articles are published in Q3 journals. Other bibliometric analyses of cancer research have highlighted the poor research output in the Arab world, with Arab countries contributing only 1.52% of total cancer publications [16] and particularly low contributions from areas affected by conflict [17].

In terms of countries, Saudi Arabia contributed to the majority of the scientific production, followed by Lebanon, Jordan, and Egypt (Figure 3). This prominence can be attributed to substantial investments in research infrastructure and supportive government policies. Saudi Arabia has developed well-equipped universities, medical centers, and laboratories, facilitating high-quality studies and collaborations. Additionally, the Saudi government prioritizes healthcare research and has implemented policies that encourage research funding and grants. These initiatives support a robust research environment, enabling significant scientific output [18]. However, after adjusting for the population size of each country, Qatar emerged as the most productive, followed by Lebanon and Saudi Arabia. Notably, some countries exhibited disparities in scientific production compared to their high CRC burden. Efforts should focus on strengthening research capacity in these countries, including training researchers, improving infrastructure, and promoting research networks. There was a lack of country-specific production in low-income countries, likely due to insufficient infrastructure and research resources (Figure 5).

The analysis of authors, affiliations, and collaborations revealed three regional clusters and highlighted some of the international collaborations with countries outside the Arab world, such as the USA and Canada. It is also noteworthy that 75.8% of the articles were single-country publications, mainly due to the fact that Saudi Arabia, the main contributor, has the majority of its production as local, as shown in Figure 4. Moreover, global journals tended to have a higher IF, which encourages cross-border collaborations and shared expertise. This underscores the need for enhanced collaboration both regionally and globally. Strengthening partnerships across Arab countries and with international researchers can help bridge gaps in research capacity, promote knowledge exchange, and improve the overall impact of CRC screening research in the Arab world.

Thematic analysis revealed that a significant proportion of the articles focused on CRC screening knowledge, awareness, and barriers, which was also reflected by the most frequent keywords. Understanding the level of knowledge and awareness about CRC screening is fundamental to informing educational and awareness campaigns and promoting preventive strategies. Also, identifying potential barriers to screening is essential to designing interventions to overcome these barriers and improve screening uptake. However, there was a lack of research focusing on other important aspects of CRC screening, with a limited number of studies investigating the implementation strategies, test selection, and cost-effectiveness of screening programs across different resource settings. These are critical areas that need to be addressed to improve the effectiveness and efficiency of CRC screening programs. Significant disparities exist in the gross domestic product (GDP) of different Arab countries, the status of their healthcare systems, and their CRC burden. For example, the current health expenditure per capita varies between 23 United States Dollars (USD) in Sudan, 62 USD in Djibouti, 63 USD in Syria, 2192 USD in the UAE, 2188 USD in Qatar, and 1533 USD in Kuwait. What is also noticeable is that countries with the lowest health expenditure have the highest out-of-pocket expenditure, which reaches 81% in Yemen and 53% in Sudan, compared to only 4.70% in Oman and 9.10% in Kuwait [19]. The cost of healthcare and lack of reimbursement can make access to screening services difficult, and studies highlighting the unmet needs of low-resource settings are crucial. Therefore, diversifying research themes in the Arab world is needed to cover all aspects of CRC screening.

Our study has certain limitations, including the restriction to English-language articles and the use of only two databases (Web of Science and Scopus) for the analysis. This may have excluded studies published in other languages or indexed in other databases. However, all the articles retrieved were available in English, and we used a comprehensive search strategy for the two databases to capture all relevant articles.

This bibliometric analysis has revealed several gaps in research on CRC screening in Arab countries. Studies on the implementation and effectiveness of different screening strategies are lacking, particularly research on the effect of screening on the incidence and mortality trends of CRC. This gap could potentially be explained by the fact that most Arab countries outside of the Gulf Cooperation Council lack a comprehensive screening strategy. For example, Qatar has implemented an organized population-based screening program, and the UAE offers population-based and opportunistic screening programs. Jordan has adopted opportunistic screening, while Kuwait, Bahrain, and Saudi Arabia have piloted screening programs, but they still lack a national program [20,21]. Therefore, more research production is needed, especially in countries with lower resources, focusing on cost-effectiveness, quality of screening services, and patient outcomes. While there are some strong regional collaborations, the clustering of alliances between certain countries can limit the impact of this research, and a broader collaboration between different Arab countries can enhance its regional and global outreach. Finally, the translation of research into practice by conducting more pilot studies of screening strategies is essential to ensure evidence-based practices.

## 5. Conclusions

In conclusion, although notable progress has been made in CRC screening research in the Arab region, significant gaps remain that need to be addressed to enhance the effectiveness of screening programs. This study highlights existing trends and gaps in CRC screening research, identifies priority areas for future studies, and guides the development of evidence-based public health policies and practices aimed at improving CRC screening uptake and outcomes in the region. Bridging these gaps will require enhanced regional and international collaborations, potentially leading to more comprehensive and effective CRC screening programs in the Arab world. Policymakers can use the findings to allocate resources and support initiatives that strengthen research capacity in low-resource Arab countries. Ultimately, these efforts will contribute to enhancing early detection of CRC and improving patient outcomes.

## Figures and Tables

**Table 1:** Overview of the main information from the collected bibliometric data on colorectal cancer screening in the Arab world from 2007 to 2023.

Description	Results
Documents	124
Sources (journals, books, etc.)	70
Keywords Plus (ID)	432
Distinct Author Keywords (DE)	242
Period	2007–2023
Document average age (years)	4.79
Annual growth rate (%)	16.49
Average citation per document	7.01
Number of references	2126
Authors	786
Authors of single-authored documents	3
Authors of multi-authored documents	713
Authors per document	6.34
International co-authorships (%)	22.58

## Data Availability

The original contributions presented in this study are included in the article/Supplementary Material. Further inquiries can be directed to the corresponding author.

## Supplementary Materials

The following supporting information can be downloaded at: www.mdpi.com/article/10.3390/ijerph22020264/s1, Figure S1: Top 10 most relevant affiliations based on the number of documents published on colorectal cancer screening in the Arab world from 2007 to 2023; Figure S2: Collaboration between the different institutions contributing to colorectal cancer screening research in the Arab world from 2007 to 2023; Figure S3: Top 10 most cited articles on colorectal cancer screening in the Arab world from 2007 to 2023; Figure S4: Co-occurrence network of Author Keywords in the colorectal cancer screening literature from the Arab world between 2007 and 2023; Table S1: Search Strategy for Web of Science database; Table S2: Search Strategy for Scopus database; Table S3: Distribution of Author Keywords by Themes (*N* = 594).

## Abbreviations

The following abbreviations are used in this manuscript:

CRC	Colorectal cancer
ASIR	Age-standardized incidence rate
ASMR	Age-standardized mortality rate
GCC	Gulf Cooperative Council
UAE	United Arab Emirates
IF	Impact factor
GDP	Gross domestic product
USD	United States dollar
