# Supplementary material for: Trends and Gaps in Colorectal Cancer Screening Research in the Arab World: A 16-Year Bibliometric Analysis (2007–2023)
Source: Int J Environ Res Public Health. 2025 Feb 12;22(2):264. doi: 10.3390/ijerph22020264 (PMC11855643; doi:10.3390/ijerph22020264)
Supplement: Supplementary file 1 [file ijerph-22-00264-s001.zip › ijerph-3411630-supplementary.pdf]

Supplementary Materials

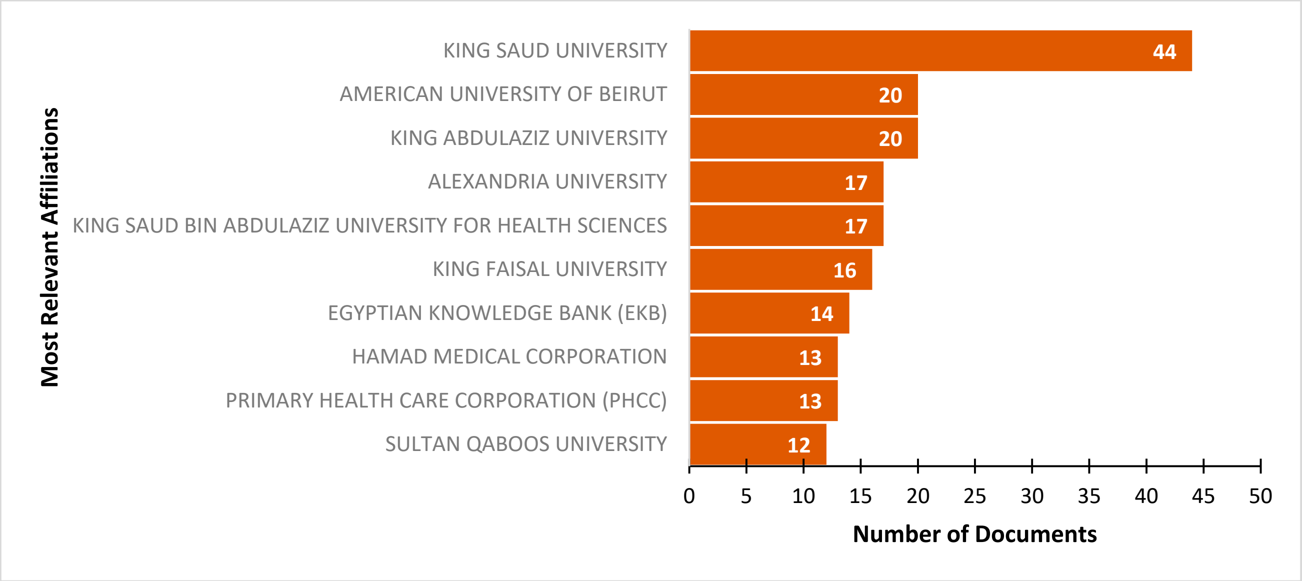

Supplementary Figure S1. Top 10 most relevant affiliations based on the number of documents published on colorectal cancer screening in the Arab world from 2007 to 2023.

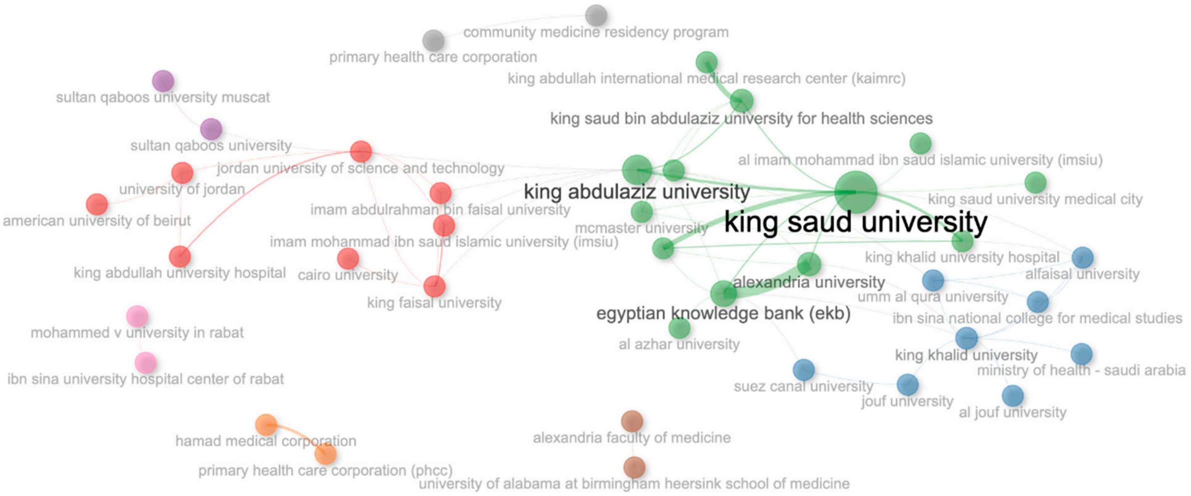

Supplementary Figure S2. Collaboration between the different institutions contributing to colorectal cancer screening research in the Arab world from 2007 to 2023. The size of the font and the diameter of the circles represent the number of publications from each institution. The colors of the circles represent the different clusters or groups of collaborating institutions. The thickness of the connecting lines indicates the strength of collaboration between the institutions.

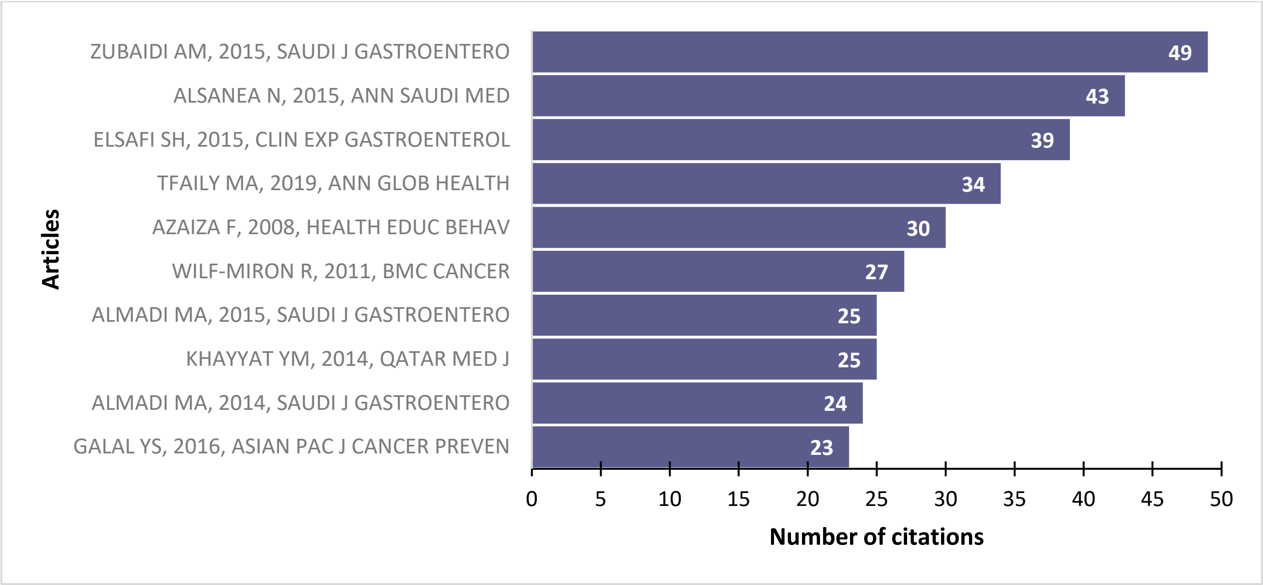

Supplementary Figure S3. Top 10 most cited articles on colorectal cancer screening in the Arab world from 2007 to 2023.

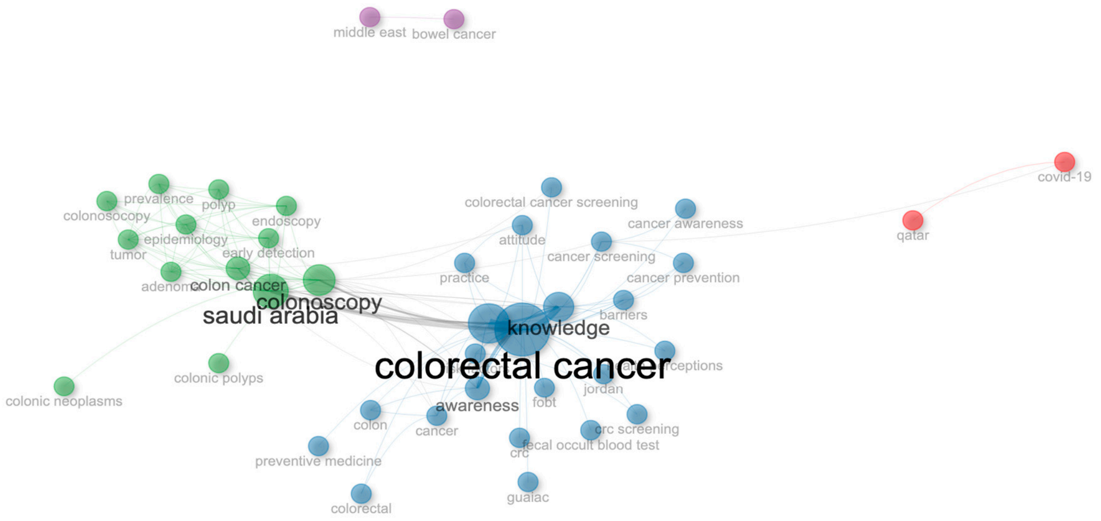

Supplementary Figure S4. Co-occurrence network of Author Keywords in the colorectal cancer screening literature from the Arab world between 2007 and 2023. The diameter of the circles and the size of the keywords reflect the frequency of each keyword's occurrence. The colors of the circles represent the different clusters or groups of related terms. The thickness of the lines connecting the keywords indicates the strength of associations between them.

A: Arab Countries:<br><br>TS=(algeria* or bahrain* or egypt* or iraq* or jordan* or kuwait* or kuweit* or lebanon or lebanese or libanaise or yemen* or aden or sanaa or UAE or Emirat* or (abu NEAR/2 dhabi) or dubai or libya* or morocco or moroccan* or ifni or (trucial NEAR/2 state) or oman* or muscat or comoros* or palestin* or gaza or ("west bank") or qatar* or katar* or quatar* or saudi* or KSA or Syria* or tunis* or (north* NEAR/2 africa*) or sudan* or djibouti* or somali* or mauritania* or MENA or EMRO or levant or (middle NEAR/2 east*) or "Near east*" or (east* NEAR/2 mediterranean) or orient or arabs or arab or arabia)
**AND**
B: Colorectal Cancer:<br><br>TS=("Colonic Cancer" OR "Colon Cancer" OR "Cancer of Colon" OR "Cancer of the Colon" OR "Colonic Neoplasm" OR "Colon Neoplasm" OR "Neoplasm of Colon" OR "Neoplasm of the Colon" OR "Colonic Tumor" OR "Colon Tumor" OR "Tumor of Colon" OR "Tumor of the Colon" OR "Rectal Cancer" OR "Rectum Cancer" OR "Cancer of Rectum" OR "Cancer of the Rectum" OR "Rectal Neoplasm" OR "Rectum Neoplasm" OR "Neoplasm of Rectum" OR "Neoplasm of the Rectum" OR "Rectal Tumor" OR "Rectum Tumor" OR "Tumor of Rectum" OR "Tumor of the Rectum" OR "Colorectal Cancer" OR "Colorectum Cancer" OR "Cancer of Colorectum" OR "Cancer of the Colorectum" OR "Colorectal Neoplasm" OR "Colorectum Neoplasm" OR "Neoplasm of Colorectum" OR "Neoplasm of the Colorectum" OR "Colorectal Tumor" OR "Colorectum Tumor" OR "Tumor of Colorectum" OR "Tumor of the Colorectum")<br>**OR**<br>C: Colorectal Cancer:<br><br>TS=(colorectal cancer* or bowel cancer* or colon cancer* or rectal cancer* or colorectal carcinoma* or bowel carcinoma* or colon carcinoma* or rectal carcinoma* or colorectal adenocarcinoma* or bowel adenocarcinoma* or colon adenocarcinoma* or rectal adenocarcinoma* or colectomy* or bowel resection or rectum resection* or colon resection* or anterior resection*)
**AND**
D: Screening:<br><br>TS = (screening) or TS = (screen) or TS = (detect) or TS = (detection) or TS=(colonoscopy) or TS=(sigmoidoscopy) or TS=(faecal occult blood test) or TS=(FOBT) OR AK = (cancer detect*) OR AK = (cancer screen*) OR AK = (early detection of cancer) OR AK = (early diagnosis of cancer)

Supplementary Table S1. Search Strategy for Web of Science database.

((TITLE-ABS-KEY ("Colonic Cancer" OR "Colon Cancer" OR "Cancer of Colon" OR "Cancer of the Colon" OR "Colonic Neoplasm" OR "Colon Neoplasm" OR "Neoplasm of Colon" OR "Neoplasm of the Colon" OR "Colonic Tumor" OR "Colon Tumor" OR "Tumor of Colon" OR "Tumor of the Colon" OR "Rectal Cancer" OR "Rectum Cancer" OR "Cancer of Rectum" OR "Cancer of the Rectum" OR "Rectal Neoplasm" OR "Rectum Neoplasm" OR "Neoplasm of Rectum" OR "Neoplasm of the Rectum" OR "Rectal Tumor" OR "Rectum Tumor" OR "Tumor of Rectum" OR "Tumor of the Rectum" OR "Colorectal Cancer" OR "Colorectum Cancer" OR "Cancer of Colorectum" OR "Cancer of the Colorectum" OR "Colorectal Neoplasm" OR "Colorectum Neoplasm" OR "Neoplasm of Colorectum" OR "Neoplasm of the Colorectum" OR "Colorectal Tumor" OR "Colorectum Tumor" OR "Tumor of Colorectum" OR "Tumor of the Colorectum") OR TITLE-ABS-KEY (colorectal AND cancer* OR bowel AND cancer* OR colon AND cancer* OR rectal AND cancer* OR colorectal AND carcinoma* OR bowel AND carcinoma* OR colon AND carcinoma* OR rectal AND carcinoma* OR colorectal AND adenocarcinoma* OR bowel AND adenocarcinoma* OR colon AND adenocarcinoma* OR rectal AND adenocarcinoma* OR colectomy* OR bowel AND resection OR rectum AND resection* OR colon AND resection* OR anterior AND resection* OR (colo* W/3 malignan*)))) OR (TITLE-ABS-KEY ("Colonic Cancer" OR "Colon Cancer" OR "Cancer of Colon" OR "Cancer of the Colon" OR "Colonic Neoplasm" OR "Colon Neoplasm" OR "Neoplasm of Colon" OR "Neoplasm of the Colon" OR "Colonic Tumor" OR "Colon Tumor" OR "Tumor of Colon" OR "Tumor of the Colon" OR "Rectal Cancer" OR "Rectum Cancer" OR "Cancer of Rectum" OR "Cancer of the Rectum" OR "Rectal Neoplasm" OR "Rectum Neoplasm" OR "Neoplasm of Rectum" OR "Neoplasm of the Rectum" OR "Rectal Tumor" OR "Rectum Tumor" OR "Tumor of Rectum" OR "Tumor of the Rectum" OR "Colorectal Cancer" OR "Colorectum Cancer" OR "Cancer of Colorectum" OR "Cancer of the Colorectum" OR "Colorectal Neoplasm" OR "Colorectum Neoplasm" OR "Neoplasm of Colorectum" OR "Neoplasm of the Colorectum" OR "Colorectal Tumor" OR "Colorectum Tumor" OR "Tumor of Colorectum" OR "Tumor of the Colorectum"))
**AND**
TITLE-ABS-KEY (screening OR screen OR detect* OR colonoscopy OR sigmoidoscopy OR ((faecal OR fecal) W/1 occult W/1 blood) OR fobt OR (cancer AND detect*) OR fit OR ((faecal OR fecal) W/1 immunochemical W/1 test) OR (cancer AND screen*) OR (early W/2 detection W/2 cancer) OR (early W/2 diagnosis W/2 cancer))
**AND**
TITLE-ABS-KEY (algeria* OR bahrain* OR egypt* OR iraq* OR jordan* OR kuwait* OR kuweit* OR lebanon OR lebanese OR libanaise OR yemen* OR aden OR sanaa OR uae OR emirat* OR (abu W/2 dhabi) OR dubai OR libya* OR morocco OR moroccan* OR ifni OR (trucial W/2 state) OR oman* OR muscat OR comoros* OR palestin* OR gaza OR ("west bank") OR qatar* OR katar* OR quatar* OR saudi* OR ksa OR syria* OR tunis* OR (north* W/2 africa*) OR sudan* OR djibouti* OR somali* OR mauritania* OR mena OR emro OR levant OR (middle W/2 east*) OR "Near east*" OR (east* W/2 mediterranean) OR orient OR arabs OR arab OR arabia)

Supplementary Table S2. Search Strategy for Scopus database.

Themes based on Author Keywords	Number, N (%)
CRC and CRC screening	**182 (30.6%)**
"Colorectal cancer" OR "CRC"	66
"Screening"	40
"Colon cancer" OR "Colonic neoplasms"	16
"Cancer" OR "Tumor"	13
"Cancer screening"	11
"Colorectal cancer screening" OR "CRC screening"	10
"Colon" OR "Colorectal"	6
"Bowel cancer"	2
"Bowel cancer screening"	2
Other	16
Awareness and attitude	**96 (16.2%)**
"Knowledge"	26
"Awareness" OR "Cancer awareness"	23
"Barriers"	8
"Attitude" OR "Attitudes"	8
"Practice" OR "Practices"	8
"Adherence"	2
"Cancer worries"	2
"Intentions"	2
"Perception"	2
Other	15
Country or population	**77 (13.0%)**
"Saudi Arabia"	28
"Jordan"	4
"Middle East"	3
"Qatar"	3
"Ethnicity"	2
"LMIC health"	2
"Oman"	2
"Population"	2
Other	31
Methods of screening	**53 (8.9%)**
"Colonoscopy"	26
"FOBT" OR "Fecal occult blood test"	8
"Endoscopy"	7
"Guaiac"	2
Other	10
Cancer epidemiology	**41 (6.9%)**
"Risk factors" OR "Risk factor"	10
"Epidemiology"	6
"Prevalence"	4
"Preventive medicine"	3
"Registry"	2
"Survey"	2
"Survival"	2
"Symptoms"	2
Other	10

Healthcare and health behavior	**41 (6.9%)**
"Health belief model"	2
"Health beliefs"	2
"Health knowledge"	2
"Health perceptions"	2
"Health promotion"	2
"Physicians"	2
"Primary healthcare"	2
"Public health"	2
Other	25
Implications of screening	**22 (3.7%)**
"Early detection" OR "Early detection of cancer"	11
"Cancer prevention" OR "Prevention"	8
Other	3
Colonoscopy findings	**19 (3.2%)**
"Colonic polyps" OR "Polyp"	8
"Adenoma"	4
Other	7
Genetic predisposition	**5 (0.8%)**
"Familial and inherited cancer" OR "Familial colorectal cancer"	2
"First-degree relatives"	1
"Genetic testing"	1
"Lynch syndrome"	1
Other keywords	**58 (9.8%)**
"COVID-19"	3
Other	55

Supplementary Table S3. Distribution of Author Keywords by Themes (*N* = 594).
